# Assessment of Family Medicine Physicians’ and Residents’ Knowledge in Managing In-Flight Medical Emergencies: A Cross-Sectional Study From Qassim, Saudi Arabia

**DOI:** 10.7759/cureus.86552

**Published:** 2025-06-22

**Authors:** Raghad Almutairi, Amel A Sulaiman

**Affiliations:** 1 Medicine, Qassim University, Qassim, SAU; 2 Family Medicine Academy, Qassim Health Cluster, Buraidah, SAU

**Keywords:** family medicine residents, in-flight, knowledge, medical emergencies, qassim region

## Abstract

Background: In-flight medical emergencies (IMEs) pose unique challenges for healthcare professionals due to the limited resources and confined environment of aircraft. Despite the potential severity of such incidents, there is limited research on the knowledge, attitudes, and practices of medical professionals regarding IMEs, particularly in specific regions such as Qassim, Saudi Arabia. Understanding these factors is crucial for improving preparedness and ensuring better outcomes for passengers experiencing medical emergencies during flights.

Methodology: This descriptive cross-sectional study was conducted among family medicine residents and primary healthcare physicians in Qassim, Saudi Arabia. A total of 155 participants were recruited from the Family Medicine Academy and affiliated primary healthcare centers. Data were collected using an online, self-administered questionnaire covering demographic information, previous training attendance, beliefs about IMEs, knowledge of managing IMEs, and attitudes and practices toward IMEs. Data were analyzed using IBM SPSS Statistics for Windows, Version 23 (Released 2015; IBM Corp., Armonk, New York), employing descriptive statistics and chi-square tests for qualitative data.

Results: The study revealed that 51.0% of participants (n = 79) had inadequate knowledge, while 49.0% (n = 76) demonstrated adequate knowledge regarding IMEs. There were no significant differences in knowledge levels across demographic factors, including gender, nationality, current qualifications, country of medical education, marital status, and monthly income. However, income below 10,000 SAR (Saudi riyal) was associated with higher rates of inadequate knowledge. Despite a willingness to volunteer during IMEs, participants expressed concerns about legal issues and their confidence in managing such emergencies. Practical responses to hypothetical IME scenarios varied widely, with correct responses ranging from 19.4% to 80.6%.

Conclusion: The findings underscore significant gaps in knowledge, attitudes, and practices among healthcare professionals in Qassim regarding IMEs. Despite a strong sense of duty to volunteer, participants expressed apprehensions about their competence and legal liabilities. Enhanced training programs and clearer guidelines are needed to improve preparedness and confidence among medical professionals, ultimately ensuring better outcomes for passengers experiencing in-flight medical emergencies.

## Introduction

Air travel is an important means of transport in the current era, and the average number of people traveling by airplane is on a rising trend. Current estimates indicate that around 2-2.5 billion passengers travel by air transportation every year [1]. People traveling through air traffic may face medical emergencies, known as in-flight medical emergencies (IMEs). A medical emergency can occur during the flight due to multiple reasons, including cabin space-related problems and atmospheric changes at higher altitudes, although most airplanes are pressurized to provide oxygen levels and pressure similar to those on the ground [2]. In some passengers, mild hypoxia may be experienced due to reduced barometric pressure inside the airplane.

In addition, passengers already affected by multiple comorbidities or in a debilitated state, as well as otherwise healthy individuals, may experience anxiety or claustrophobia during turbulence or the acceleration phase of the flight. Passengers, as well as staff on the airplane, may have a higher risk of medical emergencies if they have a prior history of respiratory, cardiac, or other diseases. Moreover, lack of sufficient sleep and disruption of the body's circadian rhythm during longer flight durations may also increase the chances of medical emergencies in pilots, cabin crew, and passengers [2]. Furthermore, personal attributes of passengers, such as increasing age, flight-related stress, security screening stress, and environmental factors inside the plane, including temperature, humidity, narrow seating, and flight delays, may increase the risk of medical emergencies during a flight [1].

Data relating to the incidence of medical emergencies during flight are minimal, mainly due to the lack of a central repository for collecting and reporting such cases [3]. Few studies have been published on the topic, and a meta-analysis reported that the global incidence of in-flight medical emergencies is estimated to be 18.2 incidents per million passengers. However, this estimate was based on low-certainty evidence and the findings of only 18 prior studies [4]. Data from airlines and ground medical consultation records suggest that the prevalence of in-flight medical emergencies may be approximately one incident per 604 flights or 24-130 incidents per one million passengers. Based on the average number of people traveling by air daily, it is estimated that in-flight medical emergencies may occur at a frequency of 260-1420 events per day worldwide [5].

Mandatory guidelines and rules to ensure safety during air travel are set for all airlines by the International Civil Aviation Organization (ICAO), an agency of the United Nations. Some of these rules are mandatory, while others are non-mandatory and may be modified by local aviation authorities according to regional needs. Mandatory guidelines for airlines to manage medical emergencies during flights include carrying sufficient medical equipment and ensuring proper staff training. Airlines are advised to equip all flights with first aid kits, emergency medical kits, and universal precaution kits. Furthermore, these guidelines recommend that cabin crew members be adequately trained to provide first aid and to use lifesaving supplies and equipment available on board [6].

Healthcare professionals traveling on a flight may volunteer to assist in the management of a medical emergency. It is important that physicians who intend to volunteer are adequately prepared to provide care. They should carry medical identification documents such as a medical license, designation card, or workplace ID as proof of their qualification to provide medical assistance. The primary role of a volunteering medical professional during an in-flight emergency is to assess the patient’s condition, gather information about available resources, and manage the situation by administering drugs or performing procedures if required.

Medical professionals are advised to begin by introducing themselves and their medical credentials to the patient and crew. Consent for treatment should be obtained from the patient. It is important to request the crew to provide all available medical resources, including first aid kits, emergency medical kits, and access to ground medical assistance. A patient history should be obtained, and vital signs should be recorded. The patient should be seated or positioned comfortably, and treatment should be administered within the physician’s scope of practice. If the patient is critically ill, the physician should recommend diverting the flight. Medical care should continue until the patient is stabilized or transferred to a healthcare facility. Finally, volunteering physicians are advised to document the encounter and report the incident to the appropriate authorities [5-8].

To date, no published study has specifically examined the preparedness of doctors to manage IMEs. As primary care doctors, who are routinely exposed to multidisciplinary cases, are expected to manage IMEs more confidently, this study aimed to assess the knowledge, attitude, and practice of primary healthcare physicians in Qassim, Saudi Arabia, in 2024, and to identify prospective areas for improving preparedness for in-flight medical emergencies in Saudi Arabia.

## Materials and methods

Study design and setting

This descriptive cross-sectional study was conducted among residents of the Qassim Family Medicine Academy (FMA) and its affiliated Primary Healthcare Centers (PHCCs) within the Qassim Health Cluster in Saudi Arabia. The study targeted all Family Medicine Academy residents (R1-R3) and primary healthcare physicians in Qassim, including both male and female participants who voluntarily agreed to participate. The study area encompassed the Family Medicine Academy and 60 accredited training centers located throughout Qassim, including major cities such as Buraydah, Unaizah, and Alrass. The study population consisted of Family Medicine residents and primary care physicians, excluding nurses, other staff, and those who refused to participate.

Sample size and sampling

The study population included 200 primary care physicians and 70 resident physicians from the Family Medicine Academy in the Qassim region. The sample size was calculated using an online sample size calculator (OpenEpi) with a 95% confidence level, a 5% margin of error, and a total population of 270, resulting in an estimated sample size of 159. A simple random sampling method was used to select primary healthcare centers, and 30 centers were chosen to achieve the desired sample size. In each center, all available physicians were considered for participation.

Data collection and procedure

Data collection was conducted using a self-administered online questionnaire developed following a comprehensive review of recent literature with similar objectives. The questionnaire, validated by previous studies, comprised two main parts: sociodemographic information and questions regarding the participants' willingness and confidence in managing in-flight medical emergencies. A pilot study with 20 participants was conducted to pretest the questionnaire, ensuring clarity and ease of understanding, and subsequent modifications were made accordingly.

A convenient non-probability sampling technique was employed to gather data from the participants. The completed questionnaires were collected through Google Forms, and data analysis was performed using the IBM SPSS Statistics for Windows, Version 23 (Released 2015; IBM Corp., Armonk, New York). Qualitative data were expressed as numbers and percentages, and the chi-square (χ²) test was used to examine differences between groups. Ethical approval for the study was obtained from the Committee of Bioethics at Qassim Health Affairs and the Ministry of Health, approval number 607/44/17076. Permission from the General Directorate of PHC (Primary Health Center) was also secured before data collection commenced. All collected data were kept confidential and used solely for research purposes.

## Results

The study included a total of 155 participants, with a mean age of 29.85 years (±5.32). The gender distribution was nearly equal, with 51% of the participants being male (n = 79). Most of the participants were Saudi nationals, comprising 89.0% (n = 138), and 44.5% of them were family medicine residents (n = 69). Among family medicine residents, most participants were in their third (41.4% (n = 29)) and first year (30.0% (n = 21)). In addition, most participants received their medical education in Saudi Arabia (87.7% (n = 136)) (Table 1).

**Table 1:** Demographic factors of Family Medicine residents and physicians, Qassim region (n = 155) N: frequency, %: percentage of frequency, mean (±SD): mean ± standard deviation of answer’s score, KSA: Kingdom of Saudi Arabia, SAR: Saudi riyal, PHC: Primary Health Center.

Variables	Categories	Frequency (n)	Percentage (%)
Age	<25 years	2	1.3%
	25-35 years	141	91.0%
	36-45 years	9	5.8%
	46-55 years	2	1.3%
	>55 years	1	0.6%
Gender	Male	79	51.0%
	Female	76	49.0%
Nationality	Saudi	138	89.0%
	Non-Saudi	17	11.0%
Current qualification	Family Medicine Consultant	8	5.2%
	Family Medicine Specialist	55	35.5%
	Family Medicine Resident	69	44.5%
	PHC physician (has no post-graduation degree in Family Medicine)	23	14.8%
If you are a family medicine resident, please select your level	R1	21	30.0%
	R2	20	28.6%
	R3	29	41.4%
Country of medical education	Saudi Arabia	136	87.7%
	Arab countries other than KSA	19	12.3%
Marital status	Single	83	53.5%
	Married	69	44.5%
	Divorced	3	1.9%
Average monthly income (SAR)?	Less than 10,000	5	3.2%
	10,000-14,999	13	8.4%
	15,000-19,999	68	43.9%
	20,000-24,999	42	27.1%
	25,000 or more	27	17.4%

The study evaluated participants' attendance at various courses and their beliefs regarding in-flight medical care. Most of the participants reported having previously attended a Basic Life Support (BLS) course (n = 153, 98.7%) and an Advanced Cardiac Life Support (ACLS) course (n = 116, 74.8%); however, a vast majority (n = 147, 94.8%) had not attended any course specifically on handling in-flight emergencies. In addition, 43.2% of the participants (n = 67) reported a limited understanding of protocols for handling in-flight emergencies, and 40.6% (n = 63) were unsure of the availability of sufficient medical resources on flights (Table 2).

**Table 2:** Participants’ attendance to different courses and their beliefs regarding in-flight medicine (n = 155) N: frequency; %: percentage of frequency.

Questions	Items		Frequency (n)	Percentage (%)
Have you previously attended a course on basic life support?		No	2	1.3%
		Yes	153	98.7%
Have you ever taken a course on advanced cardiac life support before?		No	39	25.2%
		Yes	116	74.8%
Have you ever taken a course on handling in-flight emergencies before?		No	147	94.8%
		Yes	8	5.2%
Do you understand the protocols for handling in-flight medical emergencies?		Not at all	55	35.5%
		A little bit	67	43.2%
		Yes, most of it	14	9.0%
		Yes, all of it	0	0.0%
		I am not sure	19	12.3%
Do you believe that the medical resources sufficient to handle in-flight medical emergencies are available on all flights?		No	34	21.9%
		Yes	58	37.4%
		Not sure	63	40.6%

Participants’ knowledge of managing in-flight medical emergencies was also assessed. Most participants (71.0%, n = 110) believed it was possible to connect with medical assistance on the ground during a flight emergency. Additionally, 61.3% (n = 95) correctly identified that oxygen levels decrease during a flight, and 40.0% (n = 62) correctly stated that humidity levels decrease. The most common types of in-flight medical emergencies reported by the participants were syncope (36.1%, n = 56), dizziness (24.5%, n = 38), and light-headedness (20.0%, n = 31). Regarding whether only licensed medical professionals should handle in-flight medical emergencies, opinions were split: 41.9% (n = 65) agreed, 41.9% (n = 65) disagreed, and 16.1% (n = 25) were uncertain (Table 3). 

**Table 3:** Knowledge of the participants in managing in-flight medical emergency cases (n = 155) *Statistically significant (p-value < 0.05). N: frequency, %: percentage of frequency.

Questions	Items	Frequency (n)	Percentage (%)
Do you think it is possible to connect with medical assistance on the ground during a medical emergency on a flight?	No	5	3.2%
	Yes	110	71.0%
	Not sure	40	25.8%
During a flight, the level of oxygen is	Decreased	95	61.3%
	Increased	38	24.5%
	I am not sure	22	14.2%
During a flight, the level of humidity is	Decreased	62	40.0%
	Increased	45	29.0%
	I am not sure	48	31.0%
Which one is the most common type of in-flight medical emergency?	Syncope	56	36.1%
	Myocardial Infarction	18	11.6%
	Stroke	8	5.2%
	light-headedness	31	20.0%
	Cardiac arrest	4	2.6%
	Dizziness	38	24.5%
In-flight medical emergencies should be handled only by a licensed medical professional	False	65	41.9%
	True	65	41.9%
	I don’t know	25	16.1%

The participants' attitudes toward managing in-flight medical emergencies revealed that nearly half, 49.0% (n = 76), would "probably" volunteer if needed, and over half, 51.0% (n = 79), would "probably" offer help even if someone else was assisting. A majority, 65.8% (n = 102), acknowledged the need for more training. Only a small proportion, 16.8% (n = 26), felt "definitely" equipped by their medical education. Awareness of medical supplies on flights was low, with more than half, 53.0% (n = 82), indicating they "probably" or "definitely" had no knowledge. Confidence in managing such emergencies was moderate, with a significant number, 38.1% (n = 59), feeling "probably" confident (Table 4).

**Table 4:** Attitude of the participants toward different in-flight medical emergency situation *Statistically significant (p-value < 0.05). N: frequency, %: percentage of frequency.

Questions	I am not sure		Definitely Yes		Probably Yes		Probably No		Definitely No	
	N	%	N	%	N	%	N	%	N	%
In case of an in-flight medical emergency, would you mention being a medical professional and offer to volunteer for management of the patient’s condition?	18	11.6	43	27.7	76	49.0	14	9.0	4	2.6
If someone is already helping, would you also offer to help by providing medical care to the patient?	11	7.1	41	26.5	79	51.0	19	12.3	5	3.2
If you are the only available medical professional on the flight, would you be willing to help even if you don’t completely understand the nature of the problem?	11	7.1	44	28.4	66	42.6	21	13.5	13	8.4
Will you be willing to help even if you are afraid about legal issues that may arise due to your assistance in the in-flight medical emergency management?	23	14.8	26	16.8	51	32.9	24	15.5	31	20.0
Do you think that you need more training to handle in-flight medical emergencies?	8	5.2	102	65.8	35	22.6	8	5.2	2	1.3
Do you think that your medical education has sufficiently equipped you with the knowledge and skills to handle in-flight medical emergencies?	17	11.0	26	16.8	47	30.3	43	27.7	22	14.2
Are you fully aware of the medical supplies present on the flight?	23	14.8	25	16.1	25	16.1	41	26.5	41	26.5
Do crew members receive adequate training for in-flight medical emergencies?	40	25.8	40	25.8	56	36.1	13	8.4	6	3.9
Do you know how on-ground medical consultants, on-board airline crew, and volunteer medical professionals collaborate to handle in-flight medical emergencies?	37	23.9	29	18.7	28	18.1	33	21.3	28	18.1
Do you feel confident enough to volunteer to manage in-flight medical emergencies?	21	13.5	26	16.8	59	38.1	27	17.4	22	14.2

The participants demonstrated strong knowledge of in-flight medical conditions and equipment. Most correctly identified key medical risks associated with air travel, including the effects of cabin pressure (81.3%, n = 126), the risk of venous thromboembolism on long-haul flights (93.5%, n = 145), and the absence of increased risk for preterm labor (75.5%, n = 117). They were also well-informed about the contents of an aircraft medical kit, with the majority correctly recognizing items such as sphygmomanometers (87.1%, n = 135), intravenous catheters (69.0%, n = 107), and injectable epinephrine/adrenaline (96.8%, n = 150). However, knowledge was mixed regarding the inclusion of a urinary catheter and laryngoscope (Table 5).

**Table 5:** The responses of the participants considering knowledge questions *Statistically significant (p-value < 0.05). N: frequency, %: percentage of frequency.

Knowledge questions	Incorrect answer		Correct answer	
	N	%	N	%
Does cabin pressure lead to a decrease in systemic oxyhemoglobin saturation?	29	18.7%	126	81.3%
The humidity in cabin air on a commercial airline flight is typically lower than in a typical ground-level building	55	35.5%	100	64.5%
The cabin pressure in a commercial airplane is maintained at sea level equivalent	100	64.5%	55	35.5%
Do patients with acute exacerbation of asthma benefit from altitude restriction	70	45.2%	85	54.8%
Passengers with recent abdominal surgery are at risk of wound dehiscence or bowel perforation with air travel	48	31.0%	107	69.0%
Long-haul flights are associated with an increased risk of venous thromboembolism	10	6.5%	145	93.5%
Air travel is associated with an increased risk of preterm labor (correct answer=False)	117	75.5%	38	24.5%
An aircraft medical kit usually includes a set of medical equipment, such as:				
Sphygmomanometer	20	12.9%	135	87.1%
Intravenous catheters	48	31.0%	107	69.0%
Urinary Catheter	78	50.3%	77	49.7%
Laryngoscope	97	62.6%	58	37.4%
Epinephrine/adrenaline injectable	5	3.2%	150	96.8%
Dextrose 50% injectable	15	9.7%	140	90.3%
Oral aspirin	17	11.0%	138	89.0%
Anticonvulsant injectable	25	16.1%	130	83.9%

The participants' practice behaviors in handling in-flight medical emergencies were assessed through scenario-based questions. In the first scenario, involving a 22-year-old male with an allergic reaction, the majority, 80.6% (n=125), correctly chose intramuscular epinephrine as the appropriate intervention. In a scenario involving a 65-year-old female experiencing acute abdominal pain, only one-third, 33.5% (n=52), correctly suggested instructing the pilot to lower the plane's altitude, while others chose less appropriate interventions. For a 65-year-old male who became unresponsive after chest pain, a moderate number, 41.3% (n=64), correctly opted to ask for the AED to be hooked up, though many selected less effective responses. In the final scenario, with an unresponsive 65-year-old male traveling alone, the correct comprehensive approach was identified by a majority, 63.9% (n=99), of participants (Table 6).

**Table 6:** The practice of the participants toward different situations *Statistically significant (p-value < 0.05). N: frequency, %: percentage of frequency, IM: intramuscular injection, AED: automated external defibrillator.

Case scenario questions	Answers	N	%
A 22-year-old male on a flight had snacks and drinks served Patient became suddenly short of breath and broke out in a full-body rash. By the time you get to the patient to examine him, his heart rate is 135 bpm, and yours feels like 155! His lips and tongue are swollen, and he can hardly breathe. Doctor, what should we do?	IM epinephrine (correct answer).	125	80.6%
	Aspirin 325 mg chewed.	24	15.5%
	Get albuterol from any passenger who has it. Administer as quickly as possible.	6	3.9%
A 65-year-old female with the sudden onset of acute abdominal pain. Her husband tells you that she has a history of chronic back pain. She has two Fentanyl patches on her chest. She hasn't had a bowel movement for three days. The plane is an hour from its destination. Doctor, what should we do?	Tell the pilot to lower the plane's altitude from 30,000 feet to 7,000 feet. (Correct answer).	52	33.5%
	She's in so much pain! Ask her husband to put another Fentanyl patch on her.	22	14.2%
	Give her 650 mg of Tylenol.	47	30.3%
	"I don't want to be sued if something goes wrong. I'm sorry, I can't help you here.	34	21.9%
A 65-year-old male is unresponsive. His wife tells you he complained of chest pain before he got diaphoretic and then lost consciousness. He has a history of panic attacks and a heart attack. Doctor, what should we do?	Ask the flight attendant to get the AED and also ask her to hook it up to the patient. (Correct answer).	30	19.4%
	Tell the flight attendant to bring you the AED so you can hook it up to the patient.	64	41.3%
	Ask the patient's wife for his nitroglycerine spray and quickly spray some into his mouth.	47	30.3%
	Use one of those cool seat-back phones for $10 a minute to page the cardiology fellow on call to ask for help.	14	9.0%
A 65-year-old male is unresponsive. He is traveling alone. Nobody witnessed anything strange before he became unconscious. Doctor, what should we do?	All of the above (Correct answer).	99	63.9%
	Ask the flight attendant to hook up the AED.	28	18.1%
	Place an IV and give an amp of D50.	15	9.7%
	Give Narcan.	6	3.9%
	Place oxygen via a nasal cannula.	7	4.5%

**Figure 1:**
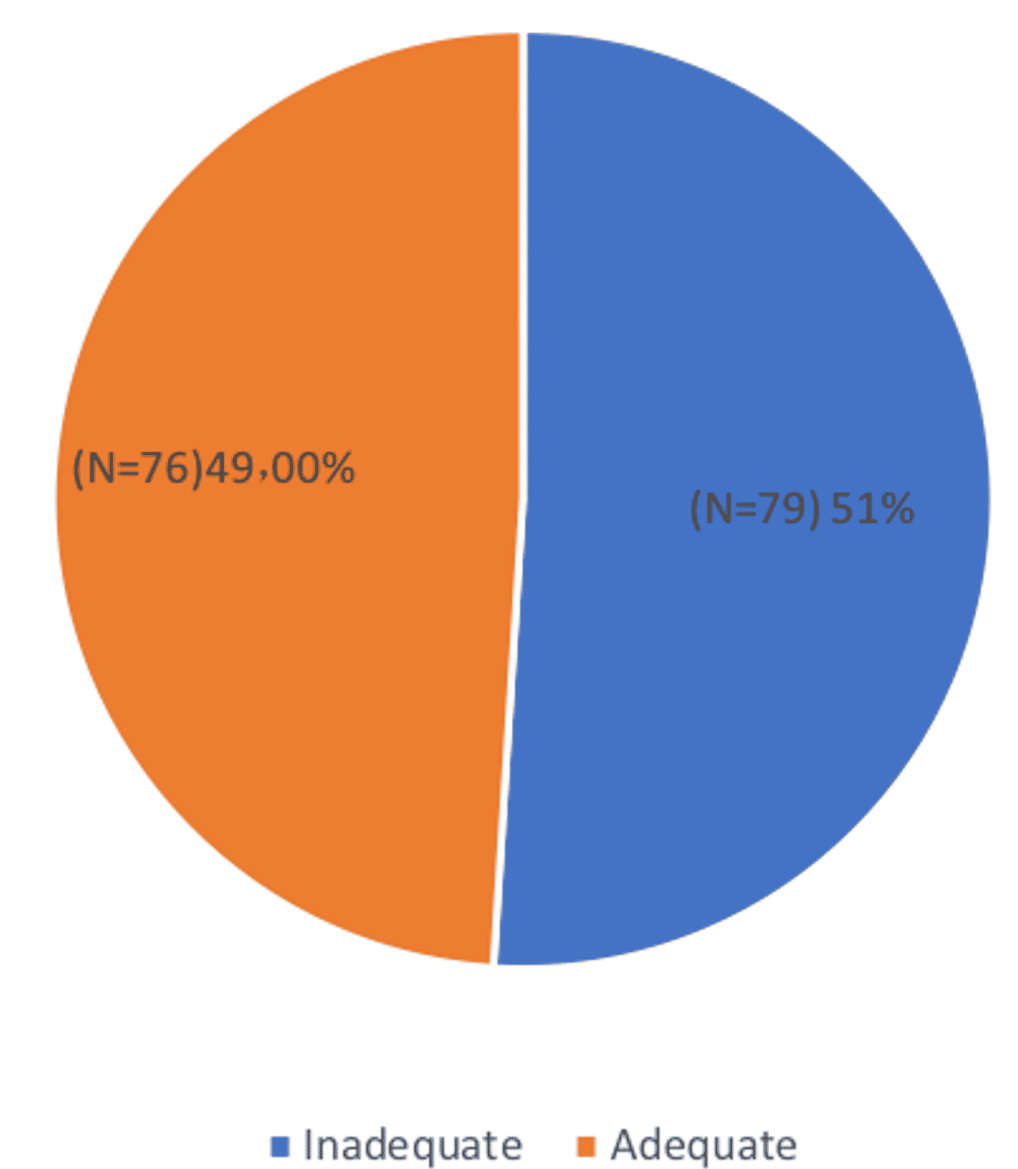
The level of knowledge among participants N: frequency, %: percentage of frequency.

The assessment of knowledge levels among participants revealed that 51.0% (n = 79) had inadequate knowledge, while 49.0% (n = 76) demonstrated adequate knowledge, indicating a nearly equal distribution of knowledge adequacy among the participants (Figure 1).

The analysis showed no significant differences in knowledge about in-flight medical emergencies across various demographic factors. Among male participants, 53.2% (n = 42) had adequate knowledge, compared to 44.7% (n = 34) of females (p = 0.294). For Saudi participants, 50.0% (n = 69) had adequate knowledge, compared to 41.2% (n = 7) of non-Saudis (p = 0.492). Family Medicine Consultants had the highest adequate knowledge at 75.0% (n = 6), while PHC physicians without a postgraduate degree had the lowest at 39.1% (n = 9) (p = 0.382). Knowledge levels across different resident levels were similar, with no significant difference based on residency year (p = 0.718). Education location and marital status showed no significant impact on knowledge, with p-values of 0.877 and 0.860, respectively. Income levels did not significantly affect knowledge, with the highest inadequate knowledge observed in those earning less than 10,000 SAR, where 100.0% (n = 5) had inadequate knowledge (p = 0.133) (Table 7).

**Table 7:** The relation between level of knowledge and demographic factors *Statistically significant (p-value < 0.05). SAR: Saudi riyal, N: frequency, %: percentage of frequency.

Variables	Categories	Knowledge				
		Inadequate		Adequate		P-value
		N	%	N	%	
Gender	Male	37	46.8%	42	53.2%	X^2^=1.1, P=0.294
	Female	42	55.3%	34	44.7%	
Nationality	Saudi	69	50.0%	69	50.0%	X^2^=0.472, P=0.492
	Non-Saudi	10	58.8%	7	41.2%	
Current qualification	Family Medicine Consultant	2	25.0%	6	75.0%	X^2^=3.06, P=0.382
	Family Medicine Specialist	28	50.9%	27	49.1%	
	Family Medicine Resident	35	50.7%	34	49.3%	
	PHC physician (has no post-graduation degree in Family Medicine)	14	60.9%	9	39.1%	
If you are a Family Medicine Resident, please select your level.	R1	12	57.1%	9	42.9%	X^2^=0.663, P=0.718
	R2	9	45.0%	11	55.0%	
	R3	14	48.3%	15	51.7%	
Country of medical education	Saudi Arabia	69	50.7%	67	49.3%	X^2^=0.024, P=0.877
	Arab countries other than KSA	10	52.6%	9	47.4%	
Marital status	Single	42	50.6%	41	49.4%	X^2^=0.302, P=0.860
	Married	35	50.7%	34	49.3%	
	Divorced	2	66.7%	1	33.3%	
Average monthly income (SAR)?	Less than 10,000	5	100.0%	0	0.0%	X^2^=4.76, P=0.133
	10,000-14,999	5	38.5%	8	61.5%	
	15,000-19,999	36	52.9%	32	47.1%	
	20,000-24,999	18	42.9%	24	57.1%	
	25,000 or more	15	55.6%	12	44.4%	

## Discussion

This study aimed to assess the knowledge, attitudes, and practices of Family Medicine residents and primary healthcare physicians in Qassim, Saudi Arabia, concerning in-flight medical emergencies. The findings offer valuable insights into the current level of preparedness among medical professionals for managing medical crises during flights.

Knowledge levels and demographic factors

The results indicated that 79 (51.0%) of the participants had inadequate knowledge, while 49.0% demonstrated adequate knowledge about in-flight medical emergencies. This near-equal distribution highlights a critical gap in the training and education of healthcare professionals concerning aviation medicine. These findings accentuate the need for improved education and awareness among healthcare professionals regarding the intricacies of in-flight medical care, aligning with the call for specialized training programs [9-11].

In the United States, physicians on board are not obligated by law to offer their assistance. However, they are ethically obliged to offer their help, and Good Samaritan laws protect volunteering medical professionals from any legal litigation [12]. Wong published the outcomes of an extensive review of existing literature about the liabilities of medical professionals to assist during in-flight emergencies and reported that the obligations of doctors as passengers on a flight are unclear, while legal protections are neither consistent nor adequate [13]. These factors contribute to the preparedness of healthcare professionals to assist during in-flight emergencies, as they may not be confident about their rights and obligations while volunteering. Caprariis et al. published the findings of a literature review aimed at elucidating the legal implications for a doctor providing emergency medical care on a commercial flight in the United States. The authors found that not all physicians may receive financial and legal support from US airlines if they face any legal litigation after providing medical care to a patient on a flight [14]. Katzer et al., from the University of California, also reported the findings of a multicenter cross-sectional study to identify the knowledge and confidence level of senior medical students regarding the management of medical emergencies during a flight. The data for this study were collected through questionnaires and responses from more than 220 fourth-year medical students who were included in the study. The average score for knowledge of the subject among the students was 64%, while students were not confident about being adequately prepared to handle in-flight medical emergencies [15].

The lack of significant differences in knowledge levels across gender, nationality, current qualifications, and resident levels underscores a systemic issue in medical education rather than demographic disparities. This finding aligns with previous studies that have identified gaps in aviation medicine training within medical curricula globally [9,11,15,16].

The study found no significant difference in knowledge levels between male and female participants (p = 0.294). This is consistent with other research that suggests gender does not significantly influence medical knowledge acquisition and retention when educational opportunities are equivalent [16-18]. However, the slight trend toward higher knowledge levels among male participants might warrant further investigation to understand any underlying causes, such as differential access to continuing medical education or variations in self-directed learning behaviors.

Participants' current qualifications showed no significant influence on their knowledge levels (p = 0.382). Notably, Family Medicine Consultants had the highest proportion of adequate knowledge (6, 75.0%), suggesting that experience and advanced training play a crucial role in acquiring and retaining critical knowledge for managing in-flight emergencies. This aligns with existing literature that emphasizes the importance of continuous professional development and practical experience in enhancing medical knowledge and skills [19].

The attitudes and practices of participants revealed a willingness to volunteer during in-flight medical emergencies, albeit with notable apprehensions about their competence and potential legal issues. Most participants (119, 76.7%) indicated they would probably or definitely volunteer to manage an in-flight medical emergency, demonstrating a strong sense of professional duty. Similar results are reported in the literature, including the study of Chatfield et al., which reported that 42% mentioned they had been asked to voluntarily assist with an in-flight medical emergency [20]. In addition, Alarifi et al. showed that 57.7% of the participants would assist during an IME [16]. However, only 16.8% felt fully confident in their ability to handle such situations, which is similar to the results of Ng et al., which showed that only 11.5% of the participating doctors felt confident to provide medical care during in-flight medical emergencies [21]. These results highlight a critical gap in self-efficacy that could impact performance during actual emergencies [22,23].

A significant majority (137, 88.4%) of participants expressed the need for more training in handling in-flight medical emergencies. This aligns with the literature emphasizing the need for specialized training programs in aviation medicine as part of medical education and continuous professional development [6,11,21]. The lack of confidence and perceived inadequate training underscore the necessity for targeted educational interventions to enhance the preparedness of healthcare professionals for in-flight medical situations [24,25].

Participants' willingness to volunteer despite fears of legal repercussions (74, 47.7% indicating they would probably or definitely help) reflects a commitment to patient care but also highlights the need for clearer guidelines and legal protections for healthcare providers in such scenarios. The Good Samaritan laws in various jurisdictions offer some protection, but their scope and application in international airspace remain ambiguous and poorly understood among medical professionals [26].

The practical responses to hypothetical in-flight medical scenarios revealed mixed adherence to best practices, with correct responses ranging from 19.4% to 80.6%. This variation indicates gaps in practical knowledge and the application of medical protocols in an aviation context. The high correct response rate for administering IM epinephrine in an anaphylactic reaction (125, 80.6%) contrasts with the lower correct response rates for other scenarios, suggesting variability in confidence and knowledge application across different emergency situations.

To address the identified knowledge gaps and improve the preparedness of healthcare professionals for in-flight medical emergencies, several recommendations can be made. Incorporating aviation medicine into medical curricula and offering regular workshops and simulations for practicing healthcare providers are essential steps. Additionally, raising awareness about the legal aspects of providing medical assistance during flights can help alleviate concerns and encourage more professionals to volunteer with confidence.

Furthermore, collaboration between healthcare institutions and airlines to develop comprehensive training programs and protocols can enhance the overall safety and efficiency of in-flight medical care. Ensuring that all medical professionals, regardless of their demographic background, have access to these educational resources is crucial for uniform preparedness.

## Conclusions

This study highlights significant gaps in the knowledge, attitudes, and practices of Family Medicine residents and primary healthcare physicians in Qassim, Saudi Arabia, regarding in-flight medical emergencies. While demographic factors such as gender, nationality, and marital status showed no significant impact on knowledge levels, there remains a critical need for enhanced training and education in this area. Addressing these gaps through targeted educational interventions and policy changes can significantly enhance the preparedness and confidence of healthcare professionals, ultimately leading to improved patient outcomes during in-flight medical emergencies.
